# Esmarch’s Method Simplified

**Published:** 1876-06

**Authors:** 


Esmarch's Method Simplified.—Dr. David Little of Rochester, N. Y., advises the application of the common roller bandage to a limb, from the distal extremity upward, elastic tubing to be applied above; when the roller is removed, the operation is found to be *absolutely bloodless.* He says that if the ordinary bandage is tightly and evenly applied it would seem to have the preference over the elastic bandage, as being more likely to force and keep out the blood from the compressed limb, simply because it is non-elastic and unyielding. It is also always at hand and easily applied.—*Am. Jour. Med. Sciences.*

